# Comparison of postural control with different customized foot orthoses on isolated subtalar arthrodesis

**DOI:** 10.1186/1757-1146-7-S1-A13

**Published:** 2014-04-08

**Authors:** E Ceccaldi

**Affiliations:** 1 Applied Podiatry College, 7 Treguel, 86000 Poitiers, France; 2 Podiatrist, Office, 35 rue Sermonoise, 77380 Combs-la-Ville, France

## Background

Studies describe subtalar and ankle arthrodesis as a factor altering the biomechanics of the foot during walking [1-3] whereas postural control appears physiological [3]. Furthermore, foot orthoses (FOs) are also recognized for their actions on dynamics [4] and balance [5] but not for their postural impact on an isolated subtalar arthrodesis (ISA). Previous studies have shown that depending on the type of FOs [6] and along the comfort felt by the subject [7], the variations induced by different FOs were significantly different. The aim of this study was to compare effects of different types of FOs on balance of patients with an ISA. Two subjects with ISA were volunteers for one session of three repeated measures: without FOs (Control), with Classical FOs (FOsC) and with Molded FOs (FOsM). After a clinical examination, these two types of FOs are custom-made including same posting. We compared postural variations through a force platform with shoes. Three modalities have been demanded at each measure: Normal stance, One-leg stance on the ISA (OnISA) and One-leg stance on the control foot (OnControl). Three data’s have been compared: Center of Pressure Area (CoP), CoP Movement (MoV) and Mean Velocity (Vel). The perception of comfort was evaluated by using previously established footwear comfort measures [7]: 100mm visual analog scale (VAS).

## Results

**Figure 1:**
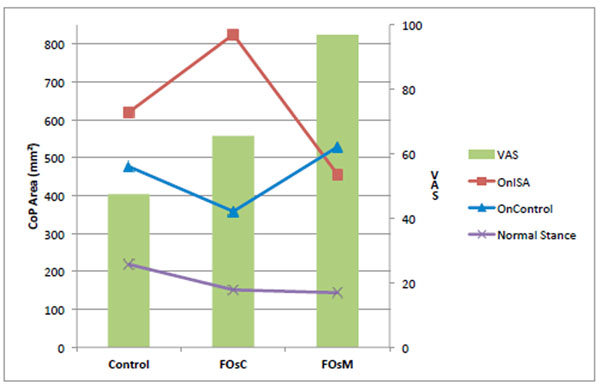
CoP Area in mm^2^

**Figure 2:**
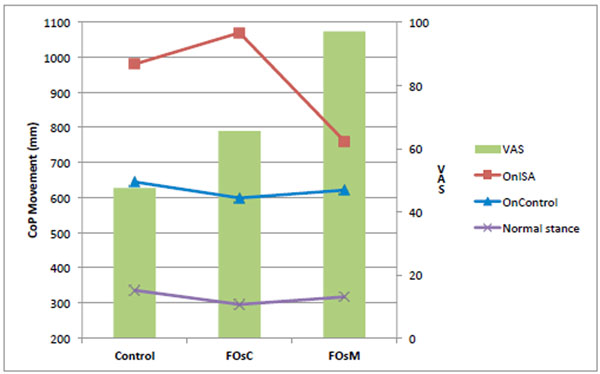
CoP Movement in mm

**Figure 3:**
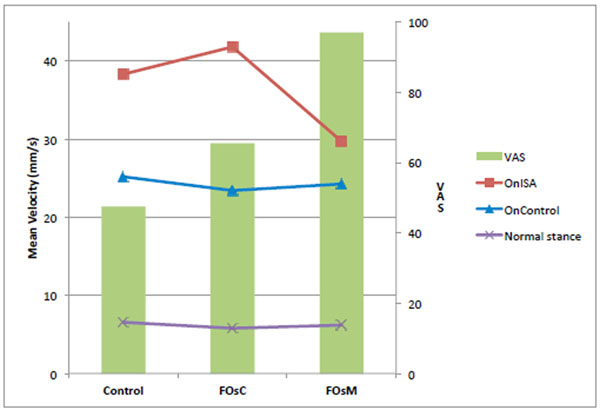
Mean Velocity in mm/s

Using the VAS, subjects didn’t feel a real comfort in their shoes without FOs (VAS=47,5mm). FOs increased VAS (>17,9mm). Thus, FOsM were perceived as significantly more comfortable than FOsC, respectively 97mm and 65,5mm. Postural assessment showed the CoP (Figure 1), the MoV (Figure 2) and the Vel (Figure 3) were improved by both FOs with Normal stance. For OnISA, the data’s indicate postural control was significantly altered by FOsC and improved by FOsM. For OnControl, postural control was more improved by FOsC.

## Conclusions

FOs induced different effects on the balance of subjects with ISA depending on orthoses type and parameters observed. FOsM appear as clearly preferable to improve postural control on an ISA. The comfort is significantly improved by FOs and much more by FOsM. The data suggests correlations between improvement of balance and perception of comfort for patients with an ISA.

## References

[B1] Wu WL Lower extremity kinematics and kinetics during level walking andstair climbing in subjects with triple arthrodesis or subtalar fusion Gait Posture 2005 21 3 263 70 10.1016/j.gaitpost.2004.02.001 15760741

[B2] Rouhani H Multi-segment foot kinematics after total ankle replacement and ankle arthrodesis during relatively long-distance gait Gait Posture 2012 36 3 561 6 10.1016/j.gaitpost.2012.05.010 22763319

[B3] Flavin R Comparison of gait after total ankle arthroplasty and ankle arthrodesis Foot Ankle Int 2013 34 10 1340 8 10.1177/1071100713490675 23669163

[B4] Telfer S Dose-response effects of customised foot orthoses on lower limb kinematics and kinetics in pronated foot type J Biomech 2013 46 9 1489 95 10.1016/j.jbiomech.2013.03.036 23631857

[B5] Gross Effects of foot orthoses on balance in older adults J Orthop Sports Phys Ther 2012 42 7 649 657 10.2519/jospt.2012.3944 22282317 PMC9477759

[B6] McPoil TG Effect of foot orthoses contour on pain perception in individuals with patellofemoral pain J Am Podiatr Med Assoc 2011 101 1 7 16 21242465 10.7547/1010007

[B7] Mills K Influence of contouring and hardness of foot orthoses on ratings of perceived comfort Med Sci Sports Exerc 2011 43 8 1507 12 10.1249/MSS.0b013e31820e783f 21233775

